# *Pneumocystis jirovecii* Pneumonia during Brentuximab Vedotin Therapy: A Case Report and Literature Review

**DOI:** 10.1155/2019/8982937

**Published:** 2019-03-26

**Authors:** Aliana Meneses Ferreira, Jessica Fernandes Ramos, Giancarlo Fatobene, Vanderson Rocha

**Affiliations:** ^1^Hematology and Bone Marrow Department, Hospital Sirio Libanes, Sao Paulo, Brazil; ^2^Infectious Diseases Department, Hospital Sirio Libanes, Sao Paulo, Brazil

## Abstract

Brentuximab vedotin (BV), an antibody drug conjugate against CD30, has been increasingly used in clinical practice, and the less common adverse events associated to the drug are not well described. Also, opportunistic infections have been reported, and data on immune reconstitution after use of BV are lacking. The authors describe a case of a 45-year-old man with Hodgkin lymphoma receiving BV as a consolidation therapy after autologous hematopoietic stem cell transplant. After nine months of consolidation with BV, the patient developed a respiratory disorder characterized by fever, chills, dyspnea, and hypoxemia, and pneumonia by *Pneumocystis jirovecii* (PJ) was confirmed by bronchoscopy with bronchoalveolar lavage. In spite of the fact that there are no specific recommendations about infectious prophylaxis in patients using the drug, we would like to draw the attention of professionals who use the medication in relation to the risk of opportunistic infections, such as pneumonia by PJ.

## 1. Introduction

*Pneumocystis jirovecii* (PJ) is an opportunistic infection known to cause life-threatening pneumonia in immunocompromised patients and is associated with substantial morbidity and mortality [1, 2]. Its incidence in patients receiving targeted therapies has not been established, although abnormalities in immune cellular function caused by these drugs may be associated with an increased risk for developing PJ pneumonia. Brentuximab vedotin (BV) is an antibody-drug conjugate against CD30 that has increasingly been used for treatment of Hodgkin lymphoma (HL) and other lymphoproliferative diseases [3].

Despite being a well-tolerated drug, opportunistic infections have been reported in patients undergoing BV therapy. PJ pneumonia, however, is rare, and only a few cases have been reported in the literature [4, 5]. Moreover, the role of the drug in the occurrence of this infection and how to best prevent it are largely unknown, while data regarding immune reconstitution after prolonged use of BV are lacking.

Herein, we report a case of PJ pneumonia in a patient with HL undergoing BV therapy, followed by a discussion on BV-related susceptibility to this infection, and suggested approaches to patients exposed to the drug.

## 2. Case Report

A 45-year-old man with no relevant medical history presented with complaints of a persistent dry cough, fever, and night sweats in August 2014. He sought urgent medical assistance a couple of times during this period and underwent empirical antibiotic therapy without improvement. A more detailed investigation using chest-computed tomography (CT) revealed thoracic lymphadenopathies; an excisional biopsy of a cervical lymph node was compatible with mixed cellularity classical HL (CD20 negative, CD3 negative, CD30 positive, CD15 negative, and PAX5 positive). Positron emission tomography-computed tomography (PET-CT) was performed for staging, which revealed involvement of the supra- and infradiaphragmatic lymph nodes, with no extranodal involvement, consistent with Ann Arbor stage IIIB disease.

The patient underwent polychemotherapy consisting of adriamycin, bleomycin, vinblastine, and dacarbazine (ABVD). After two cycles, an interim PET-CT scan was negative; he underwent a total of six cycles of ABVD treatment; nevertheless, the posttreatment PET-CT scan remained negative.

Unfortunately, five months later, in January 2015, a screening CT scan revealed iliac and retroperitoneal lymphadenopathy. A new biopsy revealed relapse of his HL. He then underwent second-line therapy with ifosfamide, carboplatin, and etoposide (ICE); however, the disease remained refractory after two cycles. A third-line therapy based on gemcitabine was successful, and hematopoietic stem cells were collected by apheresis in April 2015. A PET-CT scan after two cycles revealed complete response (Deauville score, 2), and the patient was referred to the authors' service for autologous hematopoietic stem cell transplant (HSCT) as consolidation therapy.

The autologous transplant was performed following a conditioning regimen with carmustine, etoposide, cytarabin and melphalan (BEAM) in May 2016. His transplant course was unremarkable, and neutrophil engraftment occurred 10 days later. A posttransplant PET-CT scan revealed persistent complete response.

Because the disease had relapsed <12 months after the completion of frontline therapy, the patient was started on BV according to the brentuximab vedotin as consolidation therapy after autologous stem cell transplantation in patients with Hodgkin's lymphoma at risk of relapse or progression (AETHERA) study, as posttransplant consolidation in July 2016 [6]. He received BV 1.8 mg/kg every three weeks, for a total of 16 doses.

In April 2017, after 10 doses of BV, the patient presented with sneezing, dry cough, and odynophagia, but without dyspnea, thoracic pain, or other associated symptoms. His workup revealed no abnormalities in whole blood count or acute inflammatory markers, and a chest CT scan was normal. Empirical treatment with levofloxacin and oseltamivir for upper respiratory tract infection was started, and he experienced partial improvement of symptoms. A polymerase chain reaction (PCR) assay for respiratory viruses was negative; therefore, oseltamivir was discontinued. Two weeks later, the patient returned to the authors' center with fever, chills, dyspnea, and asthenia. His physical examination was remarkable for a respiratory rate of 28 breaths/min, hypoxemia (arterial oxygen partial pressure 69 mmHg), and crackles on pulmonary auscultation. No adenopathies were observed.

**Figure 1:**
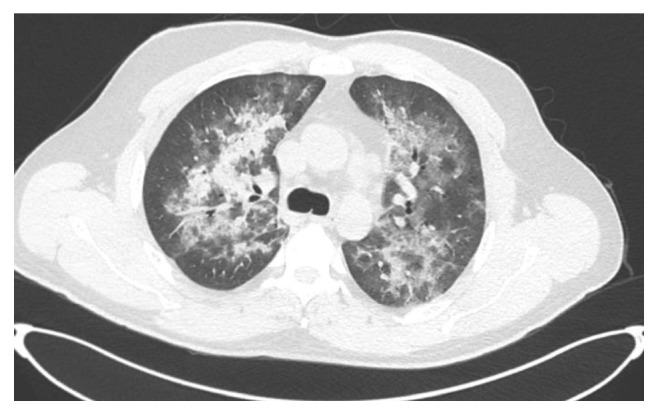
A chest-computed tomography scan revealing areas of parenchymal consolidation and diffuse ground-glass opacities.

On admission, a repeat workup revealed a hemoglobin level of 10.8 g/dL (108 g/L), leucocytes 4670/mm^3^ (neutrophils 3540/mm^3^ and lymphocytes 650/mm^3^), a platelet count of 105,000/mm^3^, and a lactate dehydrogenase level of 740 U/L (normal range, 240–480 U/L). A chest CT scan revealed areas of parenchymal consolidation and diffuse ground-glass opacities in both lungs, with predominantly peribronchovascular distribution in the upper and middle fields (Figure 1). He was started on empirical therapy with piperacillin-tazobactam (4.5 g every 6 h), oseltamivir (75 mg twice per day), and trimethoprim/sulfamethoxazole (trimethoprim 17 mg/kg/day). A bronchoscopy with bronchoalveolar lavage was performed, which was positive for PJ according to toluidine blue staining and to PCR analysis.

Prophylaxis against pneumocystis was initiated in the patient at the time of neutrophilic engraftment after the transplant in June 2016 according to institutional guidelines. However, the patient had stopped it on his own due to gastrointestinal adverse effects for the preceding two months. The last CD4 lymphocyte count available from the patient in March 2017 was 317 cells/mm^3^.

After a 21-day course of treatment with trimethoprim/sulfamethoxazole, the patient experienced full clinical recovery and was restarted on prophylaxis.

## 3. Discussion

BV is a monoclonal antibody conjugated to the cytotoxic agent monomethyl auristatin-E, targeting CD30-positive cells [7]. It was approved by the United States Food and Drug Administration (FDA) in 2011 for treatment of relapsed or refractory classical HL and anaplastic large-cell lymphoma. In 2015, the FDA expanded its indication as consolidation treatment following autologous HSCT in patients with classical HL at high risk for relapse or progression, based on the results of phase III of the AETHERA trial [6, 8].

It is not well established how BV affects the immune system, aside from its direct tumor cell cytotoxicity. Normal expression of CD30 is highly restricted to a relatively small population of activated B and T cells, and a small portion of eosinophils, suggesting that the use of BV represents a selective treatment strategy with limited interference in immune function [3]. However, we were unable to find data regarding immune abnormalities and immune reconstitution in patients treated with BV. Therefore, the risk for opportunistic infections in this population remains unclear, which necessitates specific antimicrobial prophylaxis during and after treatment.

Despite the warning on the drug label about the risk for serious and/or opportunistic infections, such as oral candidiasis and PJ pneumonia, no specific prophylaxis is recommended. Nevertheless, it is suggested that patients should be carefully monitored during treatment for the emergence of these conditions. In phase III trials of the drug, it was reported in the context of postautologous HSCT that standard international guidelines for infection prophylaxis, including PJ pneumonia, were followed [6].

Since the initial studies, BV has been increasingly used to treat patients with lymphoproliferative diseases and other medical conditions (e.g., graft versus host disease) in clinical trial settings. Because its use has become more frequent, adverse events have been reported with increasing consistency. The most common adverse events reported to date, typically grade 1 or 2 in severity, have been fatigue, pyrexia, diarrhea, nausea, neutropenia, and peripheral neuropathy. Although upper respiratory infections occur in a proportion of patients, few cases of pneumonia have been reported [7].

To our knowledge, only two cases of PJ pneumonia possibly related to BV use have been published. In a report published in 2012, involving 25 heavily pretreated patients with relapsed CD30-positive HL after allogeneic HSCT who received BV, infectious adverse events of at least grade 3 were reported in six (24%) patients. One of these patients experienced a grade 3 respiratory infection—presumably PJ pneumonia—but without laboratory confirmation [4]. The second case was reported in a cohort of 11 patients with diffuse large B-cell lymphoma treated with frontline BV-rituximab, cyclophosphamide, doxorubicin, and prednisone therapy; however, the diagnostic method was not described [5]. Unlike these cases, the diagnosis of pneumocytosis in our case was based on two laboratory methods, including toluidine blue stain, with a better positive predictive value, leaving no doubt about the diagnosis. Additionally, there is no information about these previously reported patients regarding use of prophylaxis at the time of diagnosis of the infection.

In the AETHERA study, the most common treatment-emergent adverse event in the BV group was peripheral sensory neuropathy. Severe infections (grade ≥ 3) were reported in 11 (7%) patients in the BV group and nine (6%) patients in the placebo group, with no cases of pneumocytosis reported. Similarly, no infections were reported in multiple articles, describing the experience with BV in many countries around the world.

Cases of progressive multifocal leukoencephalopathy (PML) have been reported since the approval of BV, which prompted the addition of a warning to the drug label highlighting this potential risk [9]. The physiopathology associated with PJ infection, similar to that of the John Cunningham polyomavirus involved in PML, is predominantly related to defects in CD4 T-cell-mediated immunity, although innate and humoral immune mechanisms may also play a role in host defense against these agents [10]. Therefore, it is plausible to hypothesize that patients undergoing BV therapy are at increased risk for both PML and PJ pneumonia.

Nevertheless, it is not possible to assert a causal relationship between the use of BV and the development of PJ pneumonia as multiple risk factors concomitantly present in these patients, such as lymphoid malignancies, multiagent chemotherapy, and HSCT, all of which are potential confounders in assessing the association between PJ infection and BV.

Therefore, given the high morbidity and potential mortality of pneumocytosis, this report raises awareness to the continuous risk for pneumocystis infection in patients receiving BV, to define the real need for prophylaxis. A global survey investigating specific cases of pneumocytosis in patients exposed to BV may clarify the actual magnitude of this potential serious adverse event.
